# Supplementary material for: Odorant Receptors Expressing and Antennal Lobes Architecture Are Linked to Caste Dimorphism in Asian Honeybee, Apis cerana (Hymenoptera: Apidae)
Source: Int J Mol Sci. 2024 Apr 1;25(7):3934. doi: 10.3390/ijms25073934 (PMC11012130; doi:10.3390/ijms25073934)
Supplement: Supplementary file 1 [file ijms-25-03934-s001.zip › ijms-2927408-supplementary.pdf]

Table S1 Summary of the RNA sequencing results from *A. cerana*.

Caste	Sample	Clean Reads	GC Content	≥Q30	Mapped Reads	Mapped Ratio
Queen	Antenna 1	25,545,147	36.50%	94.30%	22,955,310	89.86%
	Antenna 2	27,899,715	34.70%	96.40%	23,671,910	84.85%
	Antenna 3	19,634,513	34.72%	91.86%	15,880,769	80.88%
	Proboscis	23,798,512	37.02%	93.53%	19,980,012	83.95%
	Thorax	22,025,773	35.47%	93.60%	20,438,429	92.97%
	Abdomen	19,485,795	37.64%	93.69%	16,618,853	85.29%
	Legs	23,100,506	40.12%	94.01%	18,879,313	81.73%
Drone	Antenna 1	23,278,546	38.93%	93.59%	21,461,548	92.19%
	Antenna 2	21,368,119	38.74%	92.07%	18,632,085	87.20%
	Antenna 3	20,957,969	38.76%	93.00%	18,558,356	88.55%
	Proboscis	24,332,414	40.10%	93.27%	21,568,689	88.64%
	Thorax	24,353,115	34.73%	92.95%	18,504,552	75.98%
	Abdomen	23,359,124	39.26%	93.03%	19,277,682	82.53%
	Legs	20,763,665	40.05%	92.20%	18,643,566	89.79%
Worker	Antenna 1	20,877,517	38.31%	93.42%	19,167,456	91.81%
	Antenna 2	28,843,136	38.16%	94.47%	26,165,052	90.72%
	Antenna 3	23,367,640	38.95%	95.68%	21,798,828	93.29%
	Proboscis	22,785,896	38.90%	92.04%	19,367,362	85.00%
	Thorax	20,544,123	35.23%	92.92%	15,349,843	74.72%
	Abdomen	19,655,062	37.15%	92.30%	16,249,932	82.68%
	Legs	21,908,379	41.54%	92.64%	18,866,613	86.12%

Note: Clean Reads: counts of clean pair-end reads; GC Content: percentage of G, C in clean data; ≥Q30%: percentage of bases with Q-score no less than Q30; Mapped Reads: counts of clean reads mapped to genome; Mapped Ratio: counts of mapped to the reference genome reads and the proportion of that in clean data.

Table S2 Summary of assembly results.

Length Range	Transcript	Unigene
200-300	35,746(11.62%)	32,336(38.66%)
300-500	28,087(9.13%)	21,442(25.64%)
500-1000	32,054(10.42%)	15,200(18.17%)
1000-2000	45,639(14.84%)	7,361(8.80%)
2000+	166,089(53.99%)	7,293(8.72%)
Total Number	307,615	83,632
Total Length	885,506,748	66,221,764
N50 Length	4,692	1,576
Mean Length	2878.62	791.82

Table S3 The average FPKM value of all genes expressed in antennae. Data showed in the table indicate the mean ± SEM. Lower case alphabets indicate the significant differences calculated by one-way ANOVA followed by Tukey's multiple comparison test ($p < 0.05$).

Sample	Log_{10} (FPKM)
QA	1.84348 ± 0.01464 a
DA	1.77613 ± 0.00725 b
WA	1.74258 ± 0.00772 b

QA: Queen antenna; DA: Drone antenna; WA: Worker antenna.

Table S4 GO enrichment results on olfactory related genes ("odorant binding" categories).

Groups	DEGs	Odorant binding						
QA vs DA	Upregulated genes	APICC_00740	APICC_01795	APICC_06104	APICC_07395	APICC_09105	APICC_01038	APICC_05964
		APICC_00976	APICC_04484	APICC_06720	APICC_08050	APICC_09654	APICC_09844	APICC_09870
		APICC_01037	APICC_04782	APICC_07022	APICC_08110	APICC_09659	APICC_07149	APICC_08685
		APICC_09673						
	Downregulated genes	APICC_02313	APICC_02322	APICC_02327	APICC_02330	APICC_03357	APICC_08009	APICC_09008
		APICC_02320	APICC_02325	APICC_02328	APICC_02331	APICC_06988	APICC_08361	APICC_09701
		APICC_03149						
WA vs DA	Upregulated genes	APICC_00582	APICC_01795	APICC_05964	APICC_07022	APICC_07149	APICC_08685	APICC_08209
		APICC_0103	APICC_04484	APICC_06720	APICC_07108	APICC_07395	APICC_09654	APICC_09673
		APICC_01038	APICC_05244	APICC_06806	APICC_07148	APICC_08110	APICC_09659	APICC_09690
		APICC_09788	APICC_09844	APICC_09870				
		APICC_10028						
	Downregulated genes	APICC_01916	APICC_02325	APICC_02328	APICC_03149	APICC_06988	APICC_07721	APICC_02391
		APICC_02320	APICC_0232					
WA vs QA	Upregulated genes	APICC_00024	APICC_02318	APICC_02325	APICC_03357	APICC_05244	APICC_06806	APICC_06988
		APICC_02313	APICC_02322	APICC_02330	APICC_03890	APICC_05246	APICC_06987	APICC_07148
		APICC_07318	APICC_08009	APICC_09788	APICC_09701	APICC_09654		
	Downregulated genes	APICC_00740	APICC_01038	APICC_07022	APICC_08050	APICC_09105	APICC_09844	APICC_09870
		APICC_01037	APICC_06720	APICC_07721				

Table S5 Detailed information on identified AcerORs.

Gene	Full-length	ORF (aa)	TMD	BLASTX best hit				
				Description	Query cover	E-value	Identity	Accession
AcerOR85b	Yes	411	8	odorant receptor 13a-like [Apis dorsata]	99%	0.0	92.65%	XP_031371304.1
AcerOR91b	Yes	411	8	odorant receptor 13a-like [Apis laboriosa]	99%	0.0	86.34%	XP_043793992.1
AcerOR117	Yes	399	8	odorant receptor 13a [Apis mellifera]	100%	0.0	95.50%	XP_026300764.1
AcerOR171	Yes	395	8	odorant receptor 24a isoform X1 [Bombus terrestris]	100%	3e-163	58.25%	XP_033183372.1
AcerOR2	Yes	478	7	odorant receptor coreceptor [Apis cerana]	100%	0.0	100.00%	NP_001315406.1
AcerOR141b	Yes	432	7	odorant receptor 141 [Apis cerana cerana]	100%	0.0	99.77%	ALR87041.1
AcerOR141a	Yes	432	7	odorant receptor 13a isoform X1 [Apis cerana]	98%	0.0	98.35%	XP_016916748.1
AcerOR88	Yes	431	7	odorant receptor 13a-like [Apis cerana]	93%	0.0	97.03%	XP_016904169.1
AcerOR34	Yes	426	7	odorant receptor 46a-like [Apis laboriosa]	91%	0.0	93.09%	XP_043803265.1
AcerOR87	Yes	414	7	odorant receptor Or2-like [Apis cerana]	100%	0.0	99.52%	XP_016904173.1
AcerOR91a	Yes	412	7	odorant receptor 13a-like [Apis laboriosa]	94%	0.0	87.44%	XP_043793992.1
AcerOR85a	Yes	402	7	odorant receptor 13a-like [Apis dorsata]	96%	0.0	92.51%	XP_031371304.1
AcerOR121	Yes	398	7	odorant receptor 30a-like [Apis cerana]	100%	0.0	99.75%	XP_016921242.1
AcerOR174	Yes	393	7	odorant receptor 4-like isoform X1 [Apis mellifera caucasica]	85%	0.0	93.77%	KAG6798728.1
AcerOR172	Yes	393	7	odorant receptor 4-like isoform X1 [Apis mellifera caucasica]	85%	0.0	91.69%	KAG6798728.1
AcerOR159	Yes	392	7	odorant receptor 13a-like [Temnothorax curvispinosus]	100%	2e-31	26.57%	XP_039311370.1
AcerOR155	Yes	392	7	odorant receptor 85b-like [Bombus affinis]	90%	2e-158	62.04%	XP_003402379.2
AcerOR160b	Yes	390	7	odorant receptor 9a-like [Apis cerana]	100%	0.0	99.49%	XP_016918120.1
AcerOR160a	Yes	389	7	odorant receptor 82a-like isoform X1 [Apis dorsata]	98%	0.0	98.69%	XP_006625152.1
AcerOR106	Yes	388	7	odorant receptor 22c-like [Apis cerana]	100%	0.0	99.23%	XP_016908637.1
AcerOR71b	Yes	379	7	odorant receptor Or1-like isoform X3 [Apis cerana]	99%	0.0	99.47%	XP_016905289.1
AcerOR149b	Yes	377	7	odorant receptor 43a-like isoform X2 [Apis mellifera]	98%	0.0	70.78%	XP_026294950.1

Gene	Full-length	ORF (aa)	TMD	BLASTX best hit				
				Description	Query cover	E-value	Identity	Accession
AcerOR149a	Yes	371	7	odorant receptor 43a-like isoform X2 [Apis mellifera]	95%	0.0	70.06%	XP_026294950.1
AcerOR156	Yes	365	7	odorant receptor 67a-like [Apis cerana]	96%	0.0	100.00%	XP_016922948.1
AcerOR151	Yes	336	7	odorant receptor 49a-like [Apis cerana]	92%	0.0	98.06%	XP_016913160.1
AcerOR19	Yes	464	6	Odorant receptor 43a [Apis cerana cerana]	99%	0.0	68.98%	PBC34545.1
AcerOR166b	Yes	436	6	odorant receptor 167 [Apis cerana cerana]	100%	0.0	99.77%	ALR81939.1
AcerOR23	Yes	433	6	odorant receptor 85b-like [Apis mellifera]	100%	0.0	91.01%	XP_006565947.2
AcerOR69	Yes	426	6	odorant receptor 46a-like [Apis dorsata]	84%	0.0	93.48%	XP_031370968.1
AcerOR166a	Yes	425	6	odorant receptor 167 [Apis cerana cerana]	98%	0.0	99.76%	ALR81939.1
AcerOR97	Yes	424	6	odorant receptor 9a-like isoform X2 [Bombus vancouverensis nearcticus]	89%	4e-163	55.87%	XP_033189868.1
AcerOR109a	Yes	421	6	odorant receptor 109 [Apis mellifera]	99%	0.0	89.02%	NP_001229917.2
AcerOR109b	Yes	421	6	odorant receptor 109 [Apis mellifera]	99%	0.0	89.26%	NP_001229917.2
AcerOR35	Yes	413	6	odorant receptor 67a-like isoform X1 [Apis cerana]	100%	0.0	100.00%	XP_028521978.1
AcerOR24	Yes	412	6	odorant receptor 22c-like [Apis laboriosa]	100%	0.0	94.90%	XP_043803306.1
AcerOR26	Yes	410	6	odorant receptor 4-like [Apis cerana]	100%	0.0	99.76%	XP_016910436.1
AcerOR79b	Yes	407	6	odorant receptor 13a-like [Apis cerana]	100%	0.0	98.53%	XP_016904176.1
AcerOR48	Yes	407	6	odorant receptor 4-like [Apis laboriosa]	99%	0.0	88.18%	XP_043803278.1
AcerOR44	Yes	406	6	odorant receptor 82a-like [Apis mellifera]	100%	0.0	86.45%	XP_003250750.1
AcerOR28a	Yes	405	6	odorant receptor 4 isoform X1 [Apis cerana]	100%	0.0	99.51%	XP_016910454.1
AcerOR28b	Yes	405	6	odorant receptor 4 isoform X1 [Apis cerana]	100%	0.0	99.75%	XP_016910454.1
AcerOR46	Yes	405	6	odorant receptor 4-like [Apis cerana]	100%	0.0	80.25%	XP_016910420.1
AcerOR79a	Yes	404	6	odorant receptor 13a-like [Apis cerana]	100%	0.0	93.56%	XP_016904176.1
AcerOR76	Yes	404	6	odorant receptor 13a-like [Apis dorsata]	100%	0.0	93.66%	XP_031371292.1
AcerOR84	Yes	402	6	odorant receptor 13a-like [Apis dorsata]	99%	0.0	80.68%	XP_031371304.1
AcerOR96b	Yes	402	6	odorant receptor 96 [Apis cerana cerana]	100%	0.0	99.75%	AQY09989.1
AcerOR74	Yes	402	6	Odorant receptor Or2 [Apis cerana cerana]	99%	0.0	100.00%	PBC27163.1

Gene	Full-length	ORF (aa)	TMD	BLASTX best hit				
				Description	Query cover	E-value	Identity	Accession
AcerOR96a	Yes	402	6	odorant receptor 13a-like [Apis cerana]	100%	0.0	99.75%	XP_016904190.1
AcerOR1	Yes	400	6	odorant receptor 30a-like [Apis cerana]	100%	0.0	99.75%	NP_001315407.1
AcerOR62b	Yes	399	6	odorant receptor 63 isoform X1 [Apis mellifera]	100%	0.0	95.99%	XP_006563549.2
AcerOR169bN	No	399	6	odorant receptor 22c-like [Apis cerana]	99%	0.0	77.50%	XP_016904924.2
AcerOR169aN	No	398	6	odorant receptor 22c-like [Apis cerana]	100%	0.0	74.56%	XP_016904923.1
AcerOR111	Yes	398	6	odorant receptor 22c-like [Apis cerana]	100%	0.0	99.75%	XP_016908625.1
AcerOR18	Yes	398	6	Odorant receptor 43a [Apis cerana cerana]	99%	0.0	99.75%	PBC34545.1
AcerOR22	Yes	398	6	odorant receptor 22c-like [Apis cerana]	98%	0.0	86.77%	XP_016904924.2
AcerOR75N	No	397	6	Odorant receptor Or2 [Apis cerana cerana]	100%	0.0	83.80%	PBC27163.1
AcerOR12	Yes	396	6	odorant receptor 4-like [Apis dorsata]	100%	0.0	84.60%	XP_006615207.1
AcerOR170	Yes	396	6	odorant receptor 22c-like [Apis cerana]	100%	0.0	98.99%	XP_016904923.1
AcerOR168C	No	394	6	odorant receptor 22c-like [Apis cerana]	99%	0.0	98.98%	XP_016904924.2
AcerOR115	Yes	394	6	odorant receptor 13a-like isoform X2 [Apis cerana]	82%	0.0	100.00%	XP_016916414.1
AcerOR11	Yes	394	6	odorant receptor 4-like [Apis cerana]	100%	0.0	100.00%	XP_016910326.1
AcerOR120	Yes	394	6	odorant receptor 49b-like [Apis florea]	97%	0.0	95.30%	XP_031773175.1
AcerOR66b	Yes	393	6	odorant receptor 65a isoform X2 [Apis mellifera]	100%	0.0	85.24%	XP_394081.6
AcerOR28c	Yes	393	6	odorant receptor 4 isoform X1 [Apis cerana]	99%	0.0	99.74%	XP_016910454.1
AcerOR107a	Yes	391	6	odorant receptor 67c-like [Apis cerana]	100%	0.0	99.49%	XP_016908627.1
AcerOR107b	Yes	391	6	odorant receptor 67c-like [Apis cerana]	100%	0.0	98.98%	XP_016908627.1
AcerOR113	Yes	389	6	odorant receptor 13a [Apis cerana]	100%	0.0	99.23%	XP_016908549.1
AcerOR163a	Yes	387	6	odorant receptor 85b-like [Apis cerana]	100%	0.0	98.97%	XP_028523343.1
AcerOR164b	Yes	387	6	odorant receptor 85b-like [Apis cerana]	100%	0.0	93.80%	XP_016915037.1
AcerOR164a	Yes	387	6	odorant receptor 85b-like [Apis cerana]	100%	0.0	97.16%	XP_016915037.1
AcerOR163b	Yes	387	6	odorant receptor 85b-like [Apis cerana]	100%	0.0	92.25%	XP_028523343.1
AcerOR118	Yes	386	6	odorant receptor 85b-like [Apis florea]	91%	0.0	94.07%	XP_012346755.2

Gene	Full-length	ORF (aa)	TMD	BLASTX best hit				
				Description	Query cover	E-value	Identity	Accession
AcerOR63	Yes	383	6	Putative odorant receptor 63a [Apis cerana cerana]	100%	6e-167	80.24%	XP_033189868.1
AcerOR142	Yes	375	6	odorant receptor Or2-like [Apis dorsata]	100%	0.0	86.06%	XP_031369417.1
AcerOR154c	Yes	374	6	Odorant receptor 43a [Apis cerana cerana]	100%	0.0	86.90%	PBC34862.1
AcerOR154b	Yes	374	6	Odorant receptor 43a [Apis cerana cerana]	100%	0.0	78.88%	PBC34862.1
AcerOR157	Yes	370	6	odorant receptor 4-like [Apis florea]	100%	0.0	86.49%	XP_031771870.1
AcerOR122	Yes	367	6	odorant receptor 4-like [Cataglyphis hispanica]	97%	8e-32	25.33%	XP_024883413.1
AcerOR154a	Yes	363	6	Odorant receptor 43a [Apis cerana cerana]	97%	0.0	78.75%	PBC34862.1
AcerOR131N	No	361	6	odorant receptor 131 isoform X1 [Apis mellifera]	100%	0.0	75.97%	XP_026300304.1
AcerOR72	Yes	353	6	odorant receptor Or1-like [Apis cerana]	99%	0.0	100.00%	XP_028520421.1
AcerOR29	Yes	351	6	odorant receptor 4 isoform X1 [Apis cerana]	97%	0.0	97.98%	XP_016910454.1
AcerOR14	Yes	351	6	odorant receptor 49b-like isoform X2 [Apis cerana]	94%	0.0	100.00%	XP_016910314.1
AcerOR25b	Yes	348	6	Odorant receptor 43a [Apis cerana cerana]	96%	0.0	97.31%	PBC34543.1
AcerOR25a	Yes	344	6	odorant receptor 22c-like [Apis cerana]	97%	0.0	99.10%	XP_016910425.1
AcerOR173N	No	344	6	odorant receptor 4-like [Apis dorsata]	98%	0.0	92.35%	XP_031368101.1
AcerOR66aC	No	343	6	odorant receptor 2a-like isoform X5 [Apis dorsata]	97%	0.0	92.84%	XP_031365757.1
AcerOR162	Yes	473	5	odorant receptor 13a-like [Apis florea]	80%	0.0	92.91%	XP_031774348.1
AcerOR15b	Yes	428	5	odorant receptor Or2-like isoform X4 [Apis cerana]	100%	0.0	99.77%	XP_016910304.1
AcerOR15a	Yes	428	5	odorant receptor Or2-like isoform X4 [Apis cerana]	100%	0.0	100.00%	XP_016910304.1
AcerOR53b	Yes	412	5	odorant receptor 53 [Apis mellifera]	100%	0.0	98.06%	NP_001229907.1
AcerOR53a	Yes	410	5	odorant receptor 53 [Apis mellifera]	100%	0.0	95.86%	NP_001229907.1
AcerOR161b	Yes	410	5	odorant receptor 13a-like [Apis florea]	100%	0.0	89.76%	XP_031774348.1
AcerOR54b	Yes	408	5	odorant receptor 4-like isoform X2 [Apis mellifera]	100%	0.0	96.33%	XP_026294763.1
AcerOR54a	Yes	408	5	odorant receptor 4-like isoform X2 [Apis mellifera]	100%	0.0	96.82%	XP_026294763.1
AcerOR62a	Yes	406	5	odorant receptor 63 isoform X1 [Apis mellifera]	100%	0.0	94.33%	XP_006563549.2
AcerOR86b	Yes	400	5	odorant receptor 30a-like isoform X1 [Apis cerana]	100%	0.0	100.00%	XP_016904181.1
AcerOR161a	Yes	397	5	odorant receptor 13a-like [Apis florea]	100%	0.0	92.70%	XP_031774348.1
AcerOR86a	Yes	394	5	odorant receptor 30a-like isoform X1 [Apis cerana]	96%	0.0	99.74%	XP_016904181.1

Gene	Full-length	ORF (aa)	TMD	BLASTX best hit				
				Description	Query cover	E-value	Identity	Accession
AcerOR114	Yes	383	5	odorant receptor 4-like [Apis mellifera]	100%	0.0	97.39%	XP_026294928.1
AcerOR167	Yes	373	5	odorant receptor 167 [Apis cerana cerana]	98%	0.0	100.00%	ALR81939.1
AcerOR71a	Yes	371	5	odorant receptor Or1-like isoform X4 [Apis cerana]	100%	0.0	99.46%	XP_016905290.1
AcerOR94	Yes	368	5	odorant receptor 13a-like [Apis cerana]	100%	0.0	100.00%	XP_016904174.1
AcerOR83	Yes	347	5	odorant receptor 13a-like isoform X2 [Apis cerana]	100%	0.0	97.41%	XP_028520896.1
AcerOR70	Yes	345	5	odorant receptor Or1-like isoform X4 [Apis cerana]	67%	6e-160	100.00%	XP_016905290.1
AcerOR21	Yes	339	5	odorant receptor 85b-like [Apis florea]	98%	0.0	91.34%	XP_031770828.1

Table S6 Detailed information on the glomeruli of queens from *A. cerana*.

Glomerulus	Volume($10^3 \mu m^3$)	RelativeVolume(%)	Area($10^3 \mu m^2$)	RelativeArea(%)	Class
AL1	17.70 ± 3.97	0.97 ± 0.20	3.92 ± 0.56	0.98 ± 0.15	3
AL2	32.27 ± 14.60	1.79 ± 0.77	5.55 ± 1.72	1.38 ± 0.340	1
AL3	10.94 ± 4.02	0.60 ± 0.22	2.80 ± 0.69	0.70 ± 0.18	2
AL4	30.35 ± 2.25	0.57 ± 0.11	2.63 ± 0.31	0.66 ± 0.08	3
AL5	20.71 ± 4.49	1.14 ± 0.26	4.13 ± 0.66	1.03 ± 0.16	1
AL6	15.28 ± 5.43	0.84 ± 0.30	3.40 ± 0.78	0.85 ± 0.19	2
AL7	15.69 ± 5.65	0.87 ± 0.32	3.69 ± 0.95	0.93 ± 0.25	2
AL8	38.36 ± 9.07	2.11 ± 0.47	6.47 ± 0.96	1.62 ± 0.26	1
AL9	16.39 ± 2.07	0.90 ± 0.11	3.69 ± 0.33	0.93 ± 0.10	2
AL10	15.56 ± 6.42	0.85 ± 0.34	3.54 ± 0.97	0.89 ± 0.25	2
AL11	21.69 ± 8.00	1.20 ± 0.45	4.42 ± 1.06	1.11 ± 0.27	2
AL12	31.42 ± 9.25	1.75 ± 0.57	5.43 ± 1.13	1.36 ± 0.30	2
AL13	17.44 ± 0.64	0.97 ± 0.04	3.68 ± 0.04	0.93 ± 0.03	4
AL14	16.72 ± 5.92	0.94 ± 0.35	3.55 ± 0.85	0.89 ± 0.23	1
AL15	23.62 ± 8.02	1.31 ± 0.47	4.59 ± 1.17	1.15 ± 0.31	1
AM1	9.70 ± 1.63	0.54 ± 0.11	2.50 ± 0.27	0.63 ± 0.07	2
AM2	18.73 ± 7.08	1.02 ± 0.37	3.95 ± 0.96	0.98 ± 0.22	2
AM3	11.20 ± 4.98	0.62 ± 0.28	2.65 ± 0.83	0.67 ± 0.21	1
AM4	11.64 ± 8.53	0.65 ± 0.48	2.71 ± 1.32	0.68 ± 0.34	1
AM5	6.58 ± 3.95	0.37 ± 0.23	1.85 ± 0.81	0.47 ± 0.21	2
AM6	23.38 ± 9.43	1.27 ± 0.49	4.74 ± 1.25	1.18 ± 0.29	4
AM7	16.89 ± 9.33	0.93 ± 0.50	3.89 ± 1.80	0.97 ± 0.42	4
AM8	8.36 ± 3.43	0.45 ± 0.17	2.33 ± 0.70	0.58 ± 0.17	2
AM9	12.79 ± 3.72	0.70 ± 0.19	3.02 ± 0.64	0.75 ± 0.15	3
AM10	16.19 ± 9.02	0.88 ± 0.48	3.50 ± 1.29	0.87 ± 0.30	1
AM11	12.98 ± 3.65	0.71 ± 0.19	3.05 ± 0.39	0.76 ± 0.11	4
AM12	13.05 ± 11.46	0.74 ± 0.67	3.08 ± 1.97	0.77 ± 0.51	4
AM13	22.53 ± 4.01	1.25 ± 0.27	4.80 ± 0.65	1.19 ± 0.17	2
AM14	13.55 ± 2.26	0.74 ± 0.11	3.16 ± 0.30	0.79 ± 0.08	2
AM15	11.40 ± 3.34	0.62 ± 0.17	3.00 ± 0.75	0.74 ± 0.17	1
AM16	8.39 ± 3.07	0.46 ± 0.16	2.24 ± 0.54	0.56 ± 0.14	2
AM17	10.42 ± 4.57	0.58 ± 0.28	2.96 ± 1.16	0.74 ± 0.30	1
AM18	26.93 ± 8.48	1.49 ± 0.50	5.20 ± 1.22	1.31 ± 0.32	4
AM19	13.54 ± 5.40	0.75 ± 0.31	3.24 ± 0.99	0.81 ± 0.25	2
AM20	10.08 ± 4.64	0.55 ± 0.24	2.56 ± 0.84	0.64 ± 0.21	1
AM21	25.08 ± 2.12	1.37 ± 0.10	4.74 ± 0.26	1.18 ± 0.09	2
AM22	22.73 ± 1.92	1.24 ± 0.07	4.70 ± 0.15	1.17 ± 0.06	1
AV1	14.64 ± 2.01	0.80 ± 0.08	3.32 ± 0.30	0.83 ± 0.07	1
AV2	14.72 ± 11.21	0.82 ± 0.63	3.16 ± 1.61	0.79 ± 0.41	2
AV3	7.63 ± 5.99	0.42 ± 0.34	2.07 ± 1.09	0.52 ± 0.28	2
AV4	16.56 ± 3.16	0.92 ± 0.20	3.76 ± 0.65	0.94 ± 0.17	3
AV5	19.75 ± 1.16	1.09 ± 0.11	4.08 ± 0.16	1.03 ± 0.05	1

Glomerulus	Volume($10^3\mu m^3$)	RelativeVolume(%)	Area($10^3\mu m^2$)	RelativeArea(%)	Class
AV6	14.43 ± 3.08	0.79 ± 0.16	3.21 ± 0.35	0.80 ± 0.08	1
AV7	22.40 ± 3.55	1.23 ± 0.19	4.34 ± 0.46	1.09 ± 0.11	1
AV8	18.33 ± 9.10	1.01 ± 0.50	3.64 ± 1.28	0.91 ± 0.31	1
LD1	28.74 ± 6.55	1.58 ± 0.33	5.56 ± 1.03	1.39 ± 0.23	1
LD2	18.93 ± 7.50	1.04 ± 0.42	4.09 ± 1.05	1.03 ± 0.27	4
LD3	7.91 ± 2.82	0.44 ± 0.15	2.40 ± 0.49	0.60 ± 0.11	1
MD1	21.40 ± 5.96	1.17 ± 0.29	4.38 ± 0.88	1.09 ± 0.21	1
MD2	11.19 ± 3.54	0.62 ± 0.20	2.82 ± 0.72	0.71 ± 0.19	2
MD3	29.53 ± 5.32	1.63 ± 0.34	5.43 ± 0.90	1.36 ± 0.25	1
MD4	8.95 ± 3.28	0.50 ± 0.17	2.38 ± 0.57	0.59 ± 0.13	2
MD5	18.85 ± 4.18	1.03 ± 0.20	4.14 ± 0.38	1.04 ± 0.09	2
P1	11.90 ± 2.77	0.66 ± 0.16	2.89 ± 0.35	0.72 ± 0.10	1
P2	7.83 ± 2.73	0.43 ± 0.15	2.16 ± 0.66	0.54 ± 0.16	1
P3	5.11 ± 2.75	0.28 ± 0.15	1.60 ± 0.63	0.40 ± 0.15	2
P4	3.86 ± 1.65	0.22 ± 0.09	1.33 ± 0.40	0.34 ± 0.10	1
P5	21.98 ± 6.42	1.20 ± 0.33	4.23 ± 0.93	1.05 ± 0.21	1
P6	4.76 ± 1.52	0.26 ± 0.08	1.60 ± 0.39	0.40 ± 0.09	1
P7	11.07 ± 4.96	0.60 ± 0.25	2.67 ± 0.70	0.67 ± 0.18	2
P8	10.78 ± 5.14	0.61 ± 0.30	2.78 ± 1.08	0.70 ± 0.28	1
P9	8.73 ± 1.50	0.49 ± 0.07	2.37 ± 0.35	0.59 ± 0.08	3
PD1	23.23 ± 7.37	1.27 ± 0.38	4.86 ± 1.20	1.21 ± 0.30	1
PD2	19.68 ± 7.74	1.07 ± 0.39	4.10 ± 1.18	1.02 ± 0.29	1
PD3	18.78 ± 8.11	1.04 ± 0.45	4.15 ± 1.37	1.04 ± 0.35	1
PD4	14.92 ± 5.23	0.82 ± 0.29	3.41 ± 0.89	0.85 ± 0.23	4
PD5	15.31 ± 2.80	0.84 ± 0.15	3.81 ± 0.49	0.95 ± 0.13	4
PD6	7.99 ± 3.18	0.44 ± 0.17	2.26 ± 0.69	0.56 ± 0.16	4
PD7	14.34 ± 7.90	0.78 ± 0.40	3.36 ± 1.21	0.85 ± 0.30	2
PL1	11.16 ± 2.88	0.61 ± 0.14	2.66 ± 0.45	0.66 ± 0.11	2
PL2	31.11 ± 8.96	1.72 ± 0.51	5.50 ± 1.32	1.38 ± 0.34	1
PL3	9.82 ± 0.86	0.54 ± 0.04	2.57 ± 0.19	0.64 ± 0.04	2
PL4	17.36 ± 4.60	0.94 ± 0.22	3.73 ± 0.58	0.93 ± 0.15	2
PL5	27.51 ± 5.40	1.52 ± 0.34	5.48 ± 0.84	1.37 ± 0.20	1
PL6	22.45 ± 4.89	1.24 ± 0.27	4.55 ± 0.78	1.14 ± 0.18	1
PL7	16.48 ± 5.74	0.91 ± 0.32	3.61 ± 1.01	0.90 ± 0.26	2
PL8	11.71 ± 4.95	0.64 ± 0.27	2.99 ± 1.05	0.75 ± 0.25	4
PL9	19.21 ± 8.66	1.06 ± 0.48	3.98 ± 1.48	0.99 ± 0.36	3
PL10	15.41 ± 7.38	0.86 ± 0.42	3.68 ± 1.02	0.92 ± 0.27	3
PL11	14.50 ± 8.35	0.80 ± 0.46	3.30 ± 1.42	0.82 ± 0.36	2
PL12	16.44 ± 5.04	0.91 ± 0.30	3.83 ± 0.87	0.96 ± 0.23	1
PL13	12.64 ± 8.09	0.68 ± 0.42	2.94 ± 1.31	0.74 ± 0.33	2
PL14	9.42 ± 0.08	0.53 ± 0.01	2.44 ± 0.07	0.62 ± 0.02	3
PM1	13.39 ± 6.49	0.75 ± 0.39	3.27 ± 0.96	0.82 ± 0.25	1
PM2	11.39 ± 7.99	0.62 ± 0.41	2.79 ± 1.28	0.70 ± 0.32	3

Glomerulus	Volume($10^3\mu m^3$)	RelativeVolume(%)	Area($10^3\mu m^2$)	RelativeArea(%)	Class
PM3	7.60 ± 0.95	0.41 ± 0.04	2.19 ± 0.17	0.55 ± 0.05	2
PM4	8.07 ± 1.65	0.44 ± 0.10	2.48 ± 0.49	0.62 ± 0.12	2
PM5	20.14 ± 3.24	1.09 ± 0.17	3.99 ± 0.41	0.99 ± 0.12	4
PM6	14.83 ± 5.48	0.81 ± 0.29	3.32 ± 0.82	0.83 ± 0.19	1
PM7	20.47 ± 9.56	1.14 ± 0.53	4.04 ± 1.46	1.01 ± 0.36	1
PM8	8.36 ± 3.08	0.46 ± 0.15	2.39 ± 0.54	0.60 ± 0.13	4
PM9	11.47 ± 3.64	0.63 ± 0.21	2.88 ± 0.62	0.72 ± 0.16	1
PM10	21.23 ± 2.91	1.17 ± 0.17	4.34 ± 0.49	1.09 ± 0.14	1
PM11	19.85 ± 4.11	1.09 ± 0.21	3.98 ± 0.52	1.00 ± 0.13	4
PM12	14.26 ± 2.19	0.78 ± 0.12	3.28 ± 0.25	0.82 ± 0.06	2
PM13	8.19 ± 3.89	0.45 ± 0.20	2.27 ± 0.68	0.57 ± 0.17	4
PM14	12.93 ± 4.77	0.71 ± 0.26	3.17 ± 0.88	0.79 ± 0.23	3
PM15	17.58 ± 9.58	0.97 ± 0.53	4.07 ± 1.94	1.01 ± 0.47	1
PM16	12.92 ± 6.08	0.70 ± 0.31	3.07 ± 0.92	0.77 ± 0.24	4
PM17	20.01 ± 11.56	1.09 ± 0.63	4.16 ± 1.85	1.03 ± 0.44	4
PM18	19.25 ± 7.35	1.05 ± 0.38	4.27 ± 1.17	1.07 ± 0.30	2
PM19	13.45 ± 5.63	0.75 ± 0.33	3.42 ± 0.93	0.86 ± 0.24	4
PM20	9.38 ± 4.70	0.52 ± 0.26	2.32 ± 0.83	0.58 ± 0.20	2
PM21	13.22 ± 8.90	0.71 ± 0.46	3.40 ± 1.78	0.84 ± 0.44	1
PM22	23.33 ± 9.14	1.28 ± 0.49	4.57 ± 1.46	1.14 ± 0.36	4
PM23	14.76 ± 8.55	0.82 ± 0.50	3.17 ± 1.34	0.80 ± 0.34	2
PM24	18.07 ± 8.57	0.98 ± 0.44	3.90 ± 1.11	0.98 ± 0.28	4
PM25	9.72 ± 3.32	0.54 ± 0.19	2.58 ± 0.67	0.64 ± 0.16	2
PM26	15.46 ± 5.36	0.86 ± 0.31	3.47 ± 0.95	0.87 ± 0.24	4
PM27	13.52 ± 8.93	0.72 ± 0.46	3.02 ± 1.42	0.76 ± 0.36	2
PM28	7.23 ± 0.53	0.39 ± 0.03	2.14 ± 0.23	0.53 ± 0.05	1
VC1	11.90 ± 4.00	0.65 ± 0.21	2.76 ± 0.66	0.69 ± 0.15	1
VC2	8.90 ± 1.95	0.49 ± 0.09	2.30 ± 0.35	0.57 ± 0.08	1
VC3	7.79 ± 3.23	0.42 ± 0.16	2.11 ± 0.58	0.53 ± 0.14	1
VC4	15.57 ± 4.52	0.86 ± 0.25	3.26 ± 0.65	0.81 ± 0.16	1
VC5	12.49 ± 4.91	0.70 ± 0.30	2.95 ± 0.87	0.74 ± 0.23	1
VC6	5.38 ± 1.64	0.29 ± 0.08	1.58 ± 0.33	0.39 ± 0.08	4
VC7	15.71 ± 4.67	0.86 ± 0.25	3.47 ± 0.69	0.87 ± 0.18	1
VC8	12.34 ± 2.65	0.68 ± 0.13	2.88 ± 0.41	0.72 ± 0.09	1
VC9	18.18 ± 4.98	1.00 ± 0.27	3.69 ± 0.69	0.92 ± 0.15	1
VC10	21.37 ± 3.82	1.17 ± 0.18	4.28 ± 0.56	1.07 ± 0.14	1
VC11	22.11 ± 3.76	1.23 ± 0.24	4.35 ± 0.59	1.10 ± 0.15	3
VC12	11.92 ± 4.34	0.66 ± 0.24	2.82 ± 0.81	0.71 ± 0.20	2
VC13	11.96 ± 5.15	0.65 ± 0.27	2.80 ± 0.86	0.70 ± 0.22	4
VC14	9.59 ± 5.08	0.53 ± 0.28	2.57 ± 1.02	0.64 ± 0.24	4

Four classes were identified according to the differences in glomerulus positioning and shape through four sets of data for each caste: (1) relatively consistent positioning and shape, (2) the shape is consistent but the positioning is inconsistent, (3) the positioning is consistent but the shape is inconsistent, (4) the shape and

positioning are inconsistent.

Table S7 Detailed information on the glomeruli of drones from *A. cerana*.

Glomerulus	Volume (10^3 μm^3)	Relative Volume (%)	Area (10^3 μm^2)	Relative Area (%)	Class
AL1	44.40 ± 6.28	2.18 ± 0.31	7.52 ± 0.57	2.07 ± 0.15	1
AL2	13.66 ± 4.13	0.68 ± 0.22	3.11 ± 0.65	0.86 ± 0.18	1
AL3	14.14 ± 2.55	0.69 ± 0.08	3.42 ± 0.35	0.94 ± 0.09	1
AL4	10.35 ± 2.74	0.51 ± 0.14	2.58 ± 0.48	0.71 ± 0.13	1
AL5	18.90 ± 5.32	0.93 ± 0.27	4.03 ± 0.76	1.11 ± 0.22	1
AL6	34.82 ± 7.31	1.69 ± 0.24	6.48 ± 1.10	1.78 ± 0.28	1
AL7	13.40 ± 2.70	0.65 ± 0.09	3.29 ± 0.32	0.90 ± 0.08	1
AL8	18.33 ± 7.61	0.94 ± 0.40	4.05 ± 1.35	1.12 ± 0.39	3
AL9	13.14 ± 5.28	0.63 ± 0.26	3.24 ± 1.00	0.88 ± 0.27	2
AL10	25.37 ± 8.70	1.24 ± 0.44	5.75 ± 1.78	1.58 ± 0.49	2
AL11	18.98 ± 2.27	0.93 ± 0.13	4.52 ± 0.85	1.24 ± 0.24	1
AM1	21.16 ± 5.07	1.03 ± 0.19	4.31 ± 0.57	1.18 ± 0.14	2
AM2	7.73 ± 2.35	0.38 ± 0.13	2.20 ± 0.46	0.61 ± 0.13	1
AM3	19.05 ± 4.70	0.92 ± 0.18	4.50 ± 0.85	1.23 ± 0.21	1
AM4	22.74 ± 2.83	1.11 ± 0.14	4.60 ± 0.16	1.27 ± 0.06	3
AM5	10.90 ± 4.58	0.53 ± 0.22	2.87 ± 0.71	0.79 ± 0.19	4
AM6	17.67 ± 13.26	0.88 ± 0.70	3.84 ± 2.17	1.06 ± 0.62	4
AM7	30.94 ± 13.73	1.45 ± 0.54	5.73 ± 1.36	1.56 ± 0.35	4
AM8	12.18 ± 2.52	0.59 ± 0.10	3.19 ± 0.52	0.88 ± 0.14	4
AM9	17.98 ± 3.18	0.88 ± 0.16	4.31 ± 0.34	1.19 ± 0.11	2
AM10	13.49 ± 2.14	0.66 ± 0.10	3.34 ± 0.24	0.92 ± 0.07	1
AM11	13.31 ± 3.66	0.66 ± 0.21	3.47 ± 0.80	0.95 ± 0.23	1
AM12	15.05 ± 5.01	0.75 ± 0.27	3.66 ± 0.93	1.01 ± 0.27	1
AM13	12.17 ± 5.09	0.61 ± 0.27	3.26 ± 1.12	0.90 ± 0.31	2
AM14	18.67 ± 7.00	0.94 ± 0.40	4.21 ± 0.90	1.16 ± 0.26	1
AM15	34.18 ± 14.26	1.68 ± 0.70	6.61 ± 1.75	1.82 ± 0.48	3
AM16	17.99 ± 6.31	0.89 ± 0.34	4.15 ± 0.88	1.14 ± 0.24	4
AM17	17.07 ± 11.53	0.88 ± 0.60	3.94 ± 2.03	1.09 ± 0.56	3
AM18	13.73 ± 1.98	0.66 ± 0.15	3.32 ± 0.37	0.91 ± 0.11	2
AM19	11.91 ± 2.28	0.62 ± 0.12	3.00 ± 0.36	0.84 ± 0.11	2
AM20	22.58 ± 11.67	1.18 ± 0.61	5.05 ± 2.05	1.41 ± 0.58	4
MGa	155.83 ± 23.04	7.63 ± 1.08	17.67 ± 1.93	4.85 ± 0.51	1
MGb	258.67 ± 40.82	12.59 ± 1.14	27.91 ± 1.87	7.66 ± 0.41	1
Gc	103.29 ± 11.47	4.91 ± 0.12	12.92 ± 0.41	3.55 ± 0.03	1
MGd	151.93 ± 28.51	7.39 ± 1.06	17.57 ± 1.85	4.83 ± 0.47	1
MV1	29.01 ± 7.25	1.44 ± 0.40	5.41 ± 0.75	1.49 ± 0.22	1
MV2	26.70 ±6.20	1.32 ± 0.37	5.24 ± 0.77	1.44 ± 0.23	2
MV3	14.77 ± 1.36	0.72 ± 0.05	3.44 ± 0.14	0.95 ± 0.05	1
MV4	18.98 ± 2.71	0.94 ± 0.18	4.22 ± 0.57	1.16 ± 0.17	2
MV5	14.58 ± 5.61	0.72 ± 0.29	3.35 ± 0.82	0.92 ± 0.22	2
MV6	23.74 ± 1.77	1.17 ± 0.10	4.53 ± 0.14	1.25 ± 0.04	1
MV7	63.46 ± 6.00	3.02 ± 0.02	9.52 ± 0.28	2.61 ± 0.02	1

Glomerulus	Volume (10^3 μm^3)	Relative Volume (%)	Area (10^3 μm^2)	Relative Area (%)	Class
PL1	16.05 ± 9.26	0.82 ± 0.48	3.65 ± 1.37	1.01 ± 0.38	2
PL2	21.46 ± 9.46	1.05 ± 0.46	4.32 ± 1.49	1.19 ± 0.41	4
PL3	24.13 ± 4.08	1.17 ± 0.12	4.75 ± 0.30	1.31 ± 0.06	4
PL4	18.83 ± 2.78	0.93 ± 0.19	4.09 ± 0.26	1.12 ± 0.09	2
PL5	17.85 ± 8.26	0.89 ± 0.41	4.22 ± 1.49	1.16 ± 0.41	1
PL6	25.50 ± 7.05	1.27 ± 0.39	5.08 ± 1.15	1.40 ± 0.32	2
PL7	28.10 ± 6.24	1.37 ± 0.26	5.40 ± 0.79	1.48 ± 0.22	1
PL8	58.37 ± 38.75	2.84 ± 1.95	8.68 ± 4.08	2.38 ± 1.11	1
PL9	21.86 ± 6.44	1.06 ± 0.27	4.60 ± 1.02	1.26 ± 0.26	2
PL10	22.20 ± 3.88	1.08 ± 0.14	4.45 ± 0.39	1.22 ± 0.09	3
PL11	53.57 ± 5.58	2.62 ± 0.24	8.88 ± 0.79	2.44 ± 0.22	1
PL12	21.21 ± 7.00	1.04 ± 0.37	4.56 ± 1.43	1.25 ± 0.40	2
PL13	24.79 ± 0.94	1.25 ± 0.09	5.15 ± 0.28	1.43 ± 0.09	4
PL14	26.33 ± 9.25	1.30 ± 0.54	5.16 ± 1.40	1.42 ± 0.41	2
PL15	32.69 ± 14.00	1.48 ± 0.57	5.95 ± 1.75	1.61 ± 0.46	1
PL16	45.15 ± 10.50	2.12 ± 0.60	7.96 ± 1.09	2.16 ± 0.31	1
PM1	15.63 ± 5.31	0.78 ± 0.29	3.76 ± 0.78	1.03 ± 0.22	3
PM2	8.33 ± 1.66	0.41 ± 0.11	2.32 ± 0.26	0.64 ± 0.08	1
PM3	8.86 ± 3.85	0.45 ± 0.21	2.61 ± 0.87	0.72 ± 0.25	2
PM4	22.08 ± 5.98	1.10 ± 0.34	4.61 ± 0.78	1.27 ± 0.23	1
PM5	11.23 ± 2.01	0.55 ± 0.09	3.09 ± 0.51	0.85 ± 0.13	4
PM6	19.50 ± 12.54	0.93 ± 0.57	4.14 ± 2.02	1.13 ± 0.54	4
PM7	14.21 ± 6.23	0.68 ± 0.27	3.22 ± 0.88	0.88 ± 0.23	2
PM8	14.34 ± 5.35	0.71 ± 0.28	3.70 ± 0.96	1.02 ± 0.27	3
PM9	29.37 ± 4.87	1.44 ± 0.22	5.63 ± 0.65	1.55 ± 0.17	1
PM10	27.46 ± 7.62	1.34 ± 0.34	5.39 ± 0.90	1.48 ± 0.25	2
PM11	16.95 ± 4.68	0.86 ± 0.26	4.16 ± 0.87	1.14 ± 0.25	2
PM12	27.16 ± 9.97	1.33 ± 0.47	5.49 ± 1.58	1.51 ± 0.43	1
PM13	11.33 ± 0.69	0.54 ± 0.01	2.91 ± 0.15	0.79 ± 0.03	2
PM14	10.38 ± 2.22	0.52 ± 0.10	2.89 ± 0.57	0.80 ± 0.15	2
PD1	40.14 ± 14.77	1.94 ± 0.66	7.45 ± 2.05	2.04 ± 0.54	1
PD2	43.47 ± 14.30	2.18 ± 0.80	7.64 ± 1.97	2.11 ± 0.57	4
PD3	47.35 ± 13.38	2.31 ± 0.66	8.72 ± 1.49	2.40 ± 0.41	4

Four classes were identified according to the differences in glomerulus positioning and shape through four sets of data for each caste: (1) relatively consistent positioning and shape, (2) the shape is consistent but the positioning is inconsistent, (3) the positioning is consistent but the shape is inconsistent, (4) the shape and positioning are inconsistent.

Table S8 Detailed information on the glomeruli of workers from *A. cerana*.

Glomerulus	Volume(10^3μm^3)	RelativeVolume(%)	Area(10^3μm^2)	RelativeArea(%)	Class
AL1	20.50 ± 9.45	0.59 ± 0.20	4.03 ± 1.17	0.65 ± 0.14	3
AL2	17.72 ± 6.86	0.54 ± 0.24	3.79 ± 1.05	0.63 ± 0.18	2
AL3	19.05 ± 5.42	0.56 ± 0.13	3.95 ± 0.84	0.65 ± 0.12	1
AL4	20.98 ± 6.21	0.63 ± 0.20	4.34 ± 0.73	0.72 ± 0.14	3
AL5	62.91 ± 27.09	1.94 ± 0.94	9.30 ± 3.19	1.55 ± 0.58	2
AL6	21.10 ± 3.92	0.64 ± 0.14	4.38 ± 0.42	0.72 ± 0.09	4
AL7	20.47 ± 5.20	0.61 ± 0.13	4.32 ± 0.63	0.71 ± 0.08	1
AL8	25.30 ± 6.44	0.77 ± 0.22	5.03 ± 0.71	0.83 ± 0.14	3
AL9	25.14 ± 6.79	0.75 ± 0.20	4.92 ± 0.86	0.81 ± 0.15	2
AL10	23.64 ± 5.61	0.72 ± 0.21	4.72 ± 0.83	0.78 ± 0.15	1
AL11	40.29 ± 5.57	1.21 ± 0.17	6.63 ± 0.55	1.09 ± 0.11	1
AL12	29.55 ± 10.73	0.87 ± 0.24	5.24 ± 1.18	0.85 ± 0.14	4
AL13	39.70 ± 18.46	1.17 ± 0.51	6.70 ± 2.24	1.10 ± 0.36	2
AL14	25.93 ± 10.71	0.76 ± 0.27	5.04 ± 1.48	0.82 ± 0.20	1
AL15	23.05 ± 6.04	0.69 ± 0.19	4.65 ± 0.64	0.77 ± 0.12	4
AL16	83.18 ± 15.27	2.48 ± 0.31	11.37 ± 1.33	1.87 ± 0.16	1
AL17	26.75 ± 7.19	0.79 ± 0.17	4.98 ± 0.71	0.82 ± 0.09	3
AL18	41.52 ± 6.61	1.24 ± 0.15	7.37 ± 0.93	1.21 ± 0.13	2
AL19	29.64 ± 4.50	0.89 ± 0.12	5.68 ± 0.56	0.93 ± 0.07	3
AL20	38.57 ± 7.85	1.16 ± 0.26	6.48 ± 0.95	1.07 ± 0.17	3
AL21	35.15 ± 12.61	1.03 ± 0.31	6.24 ± 1.56	1.02 ± 0.21	4
AL22	23.18 ± 4.83	0.69 ± 0.08	4.69 ± 0.51	0.77 ± 0.04	1
AL23	39.73 ± 6.13	1.18 ± 0.08	6.58 ± 0.68	1.08 ± 0.05	2
AL24	25.52 ± 2.81	0.75 ± 0.12	4.86 ± 0.42	0.79 ± 0.11	4
AM1	16.14 ± 3.47	0.49 ± 0.12	3.60 ± 0.68	0.59 ± 0.11	2
AM2	19.51 ± 6.54	0.58 ± 0.17	4.17 ± 0.81	0.68 ± 0.12	2
AM3	29.84 ± 1.64	0.90 ± 0.06	5.59 ± 0.21	0.92 ± 0.05	1
AM4	23.55 ± 2.44	0.71 ± 0.09	4.67 ± 0.42	0.77 ± 0.07	1
AM5	23.27 ± 2.24	0.70 ± 0.09	4.74 ± 0.47	0.78 ± 0.07	1
AM6	30.69 ± 16.01	0.88 ± 0.36	5.60 ± 1.70	0.91 ± 0.20	3
AM7	14.46 ± 3.45	0.43 ± 0.09	3.45 ± 0.51	0.57 ± 0.07	1
AM8	36.76 ± 9.35	1.09 ± 0.21	6.54 ± 1.25	1.07 ± 0.16	3
AM9	38.68 ± 8.89	1.15 ± 0.23	6.31 ± 0.96	1.04 ± 0.15	3
AM10	26.43 ± 9.22	0.81 ± 0.31	5.41 ± 1.04	0.90 ± 0.21	2
AM11	27.65 ± 8.32	0.82 ± 0.24	5.37 ± 1.06	0.88 ± 0.15	2
AM12	10.75 ± 2.79	0.33 ± 0.11	2.72 ± 0.56	0.45 ± 0.11	1
AM13	22.67 ± 11.34	0.68 ± 0.35	4.59 ± 1.66	0.75 ± 0.28	2
AM14	15.15 ± 4.18	0.47 ± 0.15	3.66 ± 0.52	0.61 ± 0.12	4
AM15	57.42 ± 8.57	1.74 ± 0.35	10.12 ± 1.47	1.66 ± 0.18	1
AM16	30.80 ± 20.81	0.93 ± 0.65	5.47 ± 1.99	0.91 ± 0.35	1
AM17	28.45 ± 18.81	0.81 ± 0.48	5.22 ± 2.44	0.84 ± 0.35	2
AM18	22.03 ± 5.49	0.65 ± 0.10	4.53 ± 0.68	0.74 ± 0.07	1

Glomerulus	Volume($10^3 \mu m^3$)	RelativeVolume(%)	Area($10^3 \mu m^2$)	RelativeArea(%)	Class
AM19	19.76 ± 2.13	0.59 ± 0.04	4.40 ± 0.20	0.73 ± 0.03	2
AV1	39.16 ± 13.28	1.18 ± 0.42	6.52 ± 1.83	1.07 ± 0.30	1
AV2	47.26 ± 10.72	1.40 ± 0.18	7.35 ± 0.86	1.21 ± 0.05	1
AV3	49.60 ± 10.59	1.48 ± 0.25	7.71 ± 0.96	1.27 ± 0.13	2
MV1	28.38 ± 2.26	0.86 ± 0.10	5.03 ± 0.29	0.83 ± 0.07	1
MV2	27.21 ± 9.86	0.81 ± 0.28	4.98 ± 1.33	0.82 ± 0.21	3
MV3	21.04 ± 5.39	0.63 ± 0.14	4.40 ± 0.58	0.72 ± 0.07	2
MV4	23.80 ± 6.71	0.71 ± 0.19	4.82 ± 1.09	0.79 ± 0.16	3
P1	15.59 ± 6.23	0.47 ± 0.21	3.34 ± 0.98	0.55 ± 0.17	1
P2	22.77 ± 7.42	0.69 ± 0.25	4.41 ± 1.09	0.73 ± 0.19	2
P3	8.04 ± 3.08	0.25 ± 0.10	2.16 ± 0.47	0.36 ± 0.09	2
P4	14.34 ± 11.68	0.45 ± 0.38	3.40 ± 2.23	0.57 ± 0.38	1
P5	13.33 ± 3.35	0.41 ± 0.13	3.10 ± 0.61	0.52 ± 0.12	1
P6	8.74 ± 3.61	0.26 ± 0.11	2.24 ± 0.62	0.37 ± 0.11	2
P7	14.71 ± 3.28	0.48 ± 0.11	3.40 ± 0.68	0.59 ± 0.13	4
P8	24.09 ± 15.31	0.77 ± 0.48	4.68 ± 2.44	0.79 ± 0.40	2
P9	9.04 ± 1.23	0.29 ± 0.04	2.48 ± 0.15	0.43 ± 0.03	1
PD1	66.69 ± 20.29	2.04 ± 0.73	9.88 ± 2.25	1.64 ± 0.41	1
PD2	29.31 ± 4.79	0.88 ± 0.21	5.68 ± 0.57	0.94 ± 0.16	4
PD3	21.43 ± 3.15	0.64 ± 0.08	4.45 ± 0.29	0.73 ± 0.06	1
PD4	22.11 ± 3.96	0.66 ± 0.11	4.55 ± 0.46	0.75 ± 0.08	3
PD5	31.27 ± 6.62	0.95 ± 0.25	5.80 ± 1.14	0.96 ± 0.22	3
PD6	40.47 ± 17.44	1.23 ± 0.56	6.89 ± 1.63	1.14 ± 0.30	4
PD7	35.27 ± 14.93	1.05 ± 0.43	6.24 ± 1.91	1.02 ± 0.29	1
PD8	14.15 ± 1.08	0.42 ± 0.05	3.38 ± 0.23	0.55 ± 0.06	2
PL1	22.74 ± 3.87	0.69 ± 0.13	4.30 ± 0.55	0.71 ± 0.11	1
PL2	22.43 ± 7.86	0.66 ± 0.20	4.29 ± 1.07	0.70 ± 0.15	1
PL3	46.09 ± 19.19	1.35 ± 0.42	7.05 ± 1.91	1.15 ± 0.24	2
PL4	31.16 ± 3.56	0.94 ± 0.10	6.15 ± 0.66	1.01 ± 0.07	4
PL5	29.34 ± 9.81	0.86 ± 0.22	5.40 ± 1.25	0.88 ± 0.15	4
PL6	58.99 ± 6.49	1.77 ± 0.21	8.95 ± 0.54	1.48 ± 0.12	1
PL7	49.29 ± 6.27	1.49 ± 0.24	7.56 ± 0.68	1.25 ± 0.14	3
PL8	36.35 ± 4.13	1.10 ± 0.16	6.56 ± 1.02	1.08 ± 0.16	1
PL9	26.57 ± 3.73	0.79 ± 0.04	5.07 ± 0.45	0.83 ± 0.04	1
PL10	27.70 ± 17.11	0.83 ± 0.54	4.92 ± 2.26	0.80 ± 0.36	4
PL11	23.89 ± 13.16	0.69 ± 0.35	4.55 ± 1.75	0.74 ± 0.26	4
PL12	29.54 ± 17.66	0.85 ± 0.44	5.13 ± 2.16	0.83 ± 0.30	4
PL13	25.00 ± 3.31	0.74 ± 0.16	4.85 ± 0.44	0.79 ± 0.10	4
PL14	22.21 ± 3.99	0.64 ± 0.17	4.25 ± 0.50	0.68 ± 0.13	2
PL15	33.73 ± 7.20	0.97 ± 0.29	6.05 ± 0.77	0.97 ± 0.19	2
PL16	32.02 ± 2.87	0.89 ± 0.01	5.82 ± 0.28	0.92 ± 0.03	1
PL17	34.55 ± 8.56	0.99 ± 0.33	5.96 ± 1.02	0.96 ± 0.23	1
PM1	21.86 ± 8.41	0.64 ± 0.19	4.18 ± 1.01	0.68 ± 0.13	1

Glomerulus	Volume($10^3 \mu m^3$)	RelativeVolume(%)	Area($10^3 \mu m^2$)	RelativeArea(%)	Class
PM2	37.63 ± 4.81	1.14 ± 0.18	6.56 ± 0.83	1.09 ± 0.16	1
PM3	18.99 ± 6.11	0.56 ± 0.13	4.15 ± 0.96	0.68 ± 0.13	4
PM4	16.53 ± 7.40	0.48 ± 0.18	3.68 ± 0.99	0.60 ± 0.13	3
PM5	32.84 ± 6.97	0.98 ± 0.14	5.94 ± 0.75	0.97 ± 0.06	3
PM6	25.04 ± 7.57	0.76 ± 0.30	4.67 ± 0.89	0.77 ± 0.19	2
PM7	39.54 ± 8.05	1.19 ± 0.26	6.44 ± 1.01	1.06 ± 0.18	1
PM8	14.95 ± 6.82	0.43 ± 0.15	3.39 ± 1.03	0.55 ± 0.14	1
PM9	32.66 ± 29.44	0.91 ± 0.71	5.19 ± 2.83	0.83 ± 0.38	3
PM10	18.09 ± 0.06	0.58 ± 0.02	3.85 ± 0.03	0.67 ± 0.01	4
PM11	10.57 ± 1.67	0.31 ± 0.09	2.62 ± 0.18	0.43 ± 0.07	1
PM12	27.78 ± 8.19	0.82 ± 0.19	5.07 ± 1.01	0.83 ± 0.13	1
PM13	24.02 ± 8.17	0.70 ± 0.16	5.20 ± 1.32	0.85 ± 0.15	1
PM14	27.77 ± 23.00	0.76 ± 0.55	5.23 ± 2.79	0.83 ± 0.37	3
PM15	17.61 ± 8.59	0.51 ± 0.20	3.68 ± 1.17	0.60 ± 0.16	3
PM16	17.65 ± 2.90	0.53 ± 0.09	3.78 ± 0.32	0.62 ± 0.06	1
PM17	24.40 ± 4.51	0.75 ± 0.21	5.01 ± 0.52	0.82 ± 0.14	4
PM18	22.82 ± 17.65	0.64 ± 0.42	4.42 ± 2.42	0.71 ± 0.33	3
PM19	32.67 ± 1.40	1.04 ± 0.01	5.96 ± 0.35	1.03 ± 0.05	1
PM20	9.51 ± 0.61	0.31 ± 0.01	2.52 ± 0.15	0.43 ± 0.02	2
PM21	27.85 ± 11.14	0.77 ± 0.22	5.09 ± 1.47	0.80 ± 0.17	2
PM22	23.11 ± 7.13	0.71 ± 0.27	4.67 ± 0.81	0.78 ± 0.17	4
PM23	29.04 ± 3.40	0.93 ± 0.13	5.67 ± 0.35	0.97 ± 0.07	4
PM24	17.20 ± 5.64	0.55 ± 0.17	3.70 ± 0.84	0.63 ± 0.13	1
PM25	19.25 ± 11.87	0.60 ± 0.39	3.87 ± 1.64	0.64 ± 0.29	2
PM26	20.65 ± 8.21	0.63 ± 0.28	4.22 ± 1.40	0.69 ± 0.25	2
PV1	58.93 ± 14.28	1.88 ± 0.48	8.23 ± 1.29	1.41 ± 0.21	1
PV2	23.45 ± 11.80	0.70 ± 0.36	4.38 ± 1.61	0.72 ± 0.28	1
PV3	17.51 ± 2.56	0.53 ± 0.11	3.81 ± 0.42	0.63 ± 0.09	3
PV4	15.28 ± 4.44	0.45 ± 0.09	3.63 ± 0.78	0.59 ± 0.09	1
VC1	24.10 ± 3.62	0.72 ± 0.07	4.36 ± 0.40	0.72 ± 0.03	1
VC2	35.91 ± 5.45	1.08 ± 0.13	5.79 ± 0.50	0.95 ± 0.06	2
VC3	51.30 ± 13.87	1.54 ± 0.41	7.46 ± 1.49	1.22 ± 0.22	1
VC4	42.04 ± 11.06	1.26 ± 0.33	6.59 ± 1.20	1.09 ± 0.20	1
VC5	33.17 ± 9.26	0.98 ± 0.22	5.42 ± 0.98	0.89 ± 0.12	3
VC6	25.00 ± 2.43	0.75 ± 0.03	4.59 ± 0.36	0.75 ± 0.02	1
VC7	27.50 ± 3.29	0.83 ± 0.09	4.87 ± 0.42	0.80 ± 0.06	2
VC8	17.39 ± 3.74	0.52 ± 0.09	3.56 ± 0.52	0.58 ± 0.07	1
VC9	36.61 ± 10.38	1.06 ± 0.18	5.87 ± 1.11	0.95 ± 0.11	2
VC10	45.15 ± 6.37	1.38 ± 0.30	7.01 ± 0.67	1.17 ± 0.18	2
VC11	38.98 ± 6.17	1.17 ± 0.17	6.24 ± 0.76	1.03 ± 0.11	1
VC12	35.31 ± 20.37	1.06 ± 0.63	5.70 ± 2.07	0.94 ± 0.37	2

Four classes were identified according to the differences in glomerulus positioning and shape through four sets of data for each caste: (1) relatively consistent positioning and shape, (2) the shape is consistent but the

positioning is inconsistent, (3) the positioning is consistent but the shape is inconsistent, (4) the shape and positioning are inconsistent.

Table S9 Primers for RT-qPCR of 33 candidate *AcerORs* DEGs in *A. cerana.*

Gene	Forward primer (5′ to 3′)	Reverse primer (5′ to 3′)
β-actin	GGCTCCCGAAGAACATCC	TGCGAAACACCGTCACCC
AcerOR11	ATGTGCGGTTTGCTGAAGA	CGAGAAGGTGCCAATGACG
AcerOR12	GGCTGCTCTTCTTGCGATAC	CCACCTTCACTGTCTGTTCTG
AcerOR15a	TAGGATGCGTGATTGGATACTG	AGTGACACCGAATACAGATGC
AcerOR18	TACTGCACGGGATCAGAATG	TTGAGAACAATCGGCACTTG
AcerOR22	AGGATGGAACAGTGAACATAGC	GCGAAGCAACACTAGCAATTAC
AcerOR28b	TGATTCCTTGCCTGCTTCAC	CAATGATGAGTTCGCTGTTAGG
AcerOR54a	TTCAGGAGGAATGTCGTATCAC	AGATTGCTGCCAAACTATATGC
AcerOR54b	TTGGCAGCAATCTTTGTTACAC	TGGTGGATGTTGTGATATGGAG
AcerOR66a	GGCAGTGGCAAAGGTGATG	CAGCAAGCATAGGCACAGTC
AcerOR74	ACGACGACGACGAGAAGATAC	ATATGGTGTCACGCTCAGAGG
AcerOR76	TGGAGGAACGACGAAGGAAC	CGAAGAAGGTGAAGACGAAGAC
AcerOR79a	GTCCGACATCATCAGGCATTG	ATGGAGCGTCTGGCAAGAG
AcerOR79b	CGACACGAAGCACGAGATAC	GTAGCCACCAACACTTGATACC
AcerOR96a	TCACCCTCTCTTATCGCATCTC	AGGCAACAGGCTCGTCTTG
AcerOR97	TGCGAAAGCGACAGAATGC	CAATCACCTGCCCAGTAAGAG
AcerOR107b	ATGCTCGTTGGTACTATGGTTC	CAATCAATCCTCCTCCTGTCAG
AcerOR109a	TGGAGAGTTAGTTGGTGTATGG	CGCTGTTGTCCTTCTTGAATAC
AcerOR113	TCATGGTGGTCGTCGTGTAC	GCTCGTTCGTGTCCTTGAATAG
AcerOR121	TGATACGCCGAGCAAGAATTG	CAAGCCATATACACAAGCACAC
AcerOR122	TGTGTATCAGGTGCGGATAATG	GCCCAATCTCGTTCAATGTTTG
AcerOR141a	TGGTCACGCACGATATACAATC	GACAGTATCCACGCCAACAC
AcerOR142	TGGCAACAAATGGAACAACTG	CGCTGGCACTGAACACTAC
AcerOR149a	GAAGCACAGCAATACGAAAGG	GCAAATGTACTTACACCGATGG
AcerOR149b	ATTGCCATTGTCTTTCA	TCGTATTGCTGTGCTTC
AcerOR154b	ACATTATTCGACTCAGCGTAGC	AGGCAGGAACACCATGAAGG
AcerOR155	ATGTTCATGCTGCTCCTTCC	TTCGTATTGATTCGCTTCCTTG
AcerOR159	ACTGTATCTGCTCCTCGTTGG	TTCCGTCAATGTGCTCAAGTC
AcerOR160b	ACACGACCTTGAGAAGACATTG	CCTCCACCGACCGATACAG
AcerOR163a	TCACGGCTGGAATGTTAATGG	AATGAACGCTTCGCAATTCG
AcerOR166b	TGATAGCGTAAGCACTACTTTG	AAGGCATCTAACACAGCAATAC
AcerOR168	ACCTGGCTTCCTGTATCCTC	CCAAACCATAACCGAAGTAACG
AcerOR170	GCTAGTGTTGCTTCGCTTATG	TTATCGTATCTTCACCGCCAAG
AcerOR174	ATGATTTGTGTTGGTCTTTGGC	TGGGCGAGTGTGATTTAAAGG

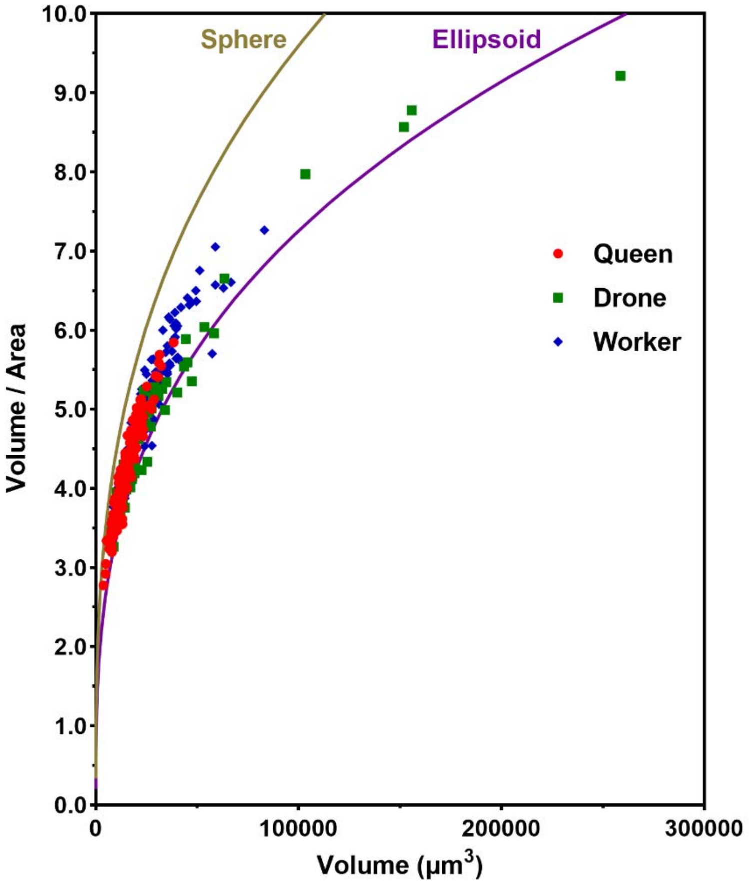

Figure S1 Quantification method on the shape of glomeruli in *A. cerana*. X axis indicate the volume of each glomerulus, Y axis indicate the Volume / Area ratio of each glomerulus, the dots near the brown line are classified into a sphere shape, while the dots near the purple line are classified into an ellipsoid shape. This method is used to classify the shape of glomeruli in our studies.

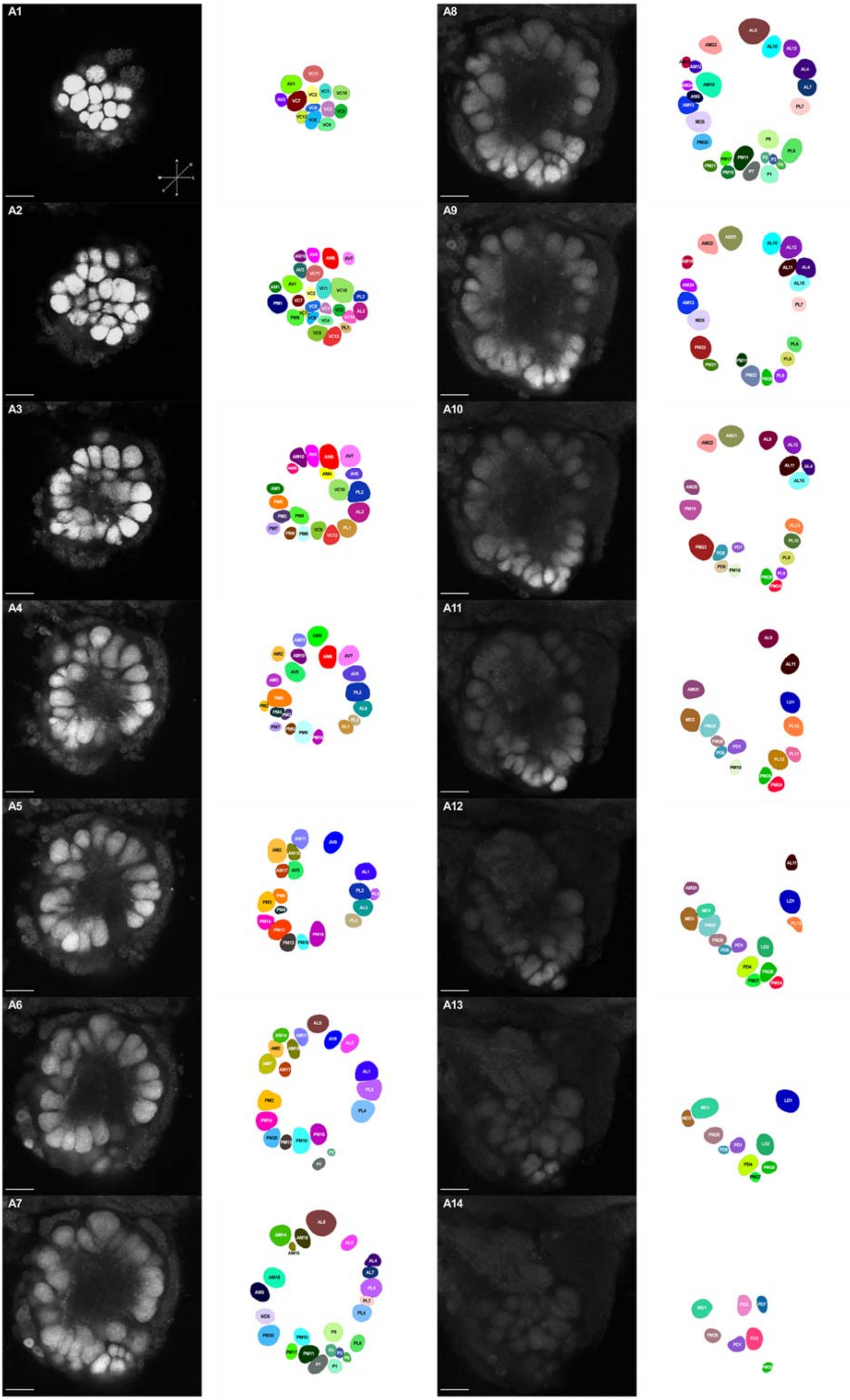
A1
A2
A3
A4
A5
A6
A7
A8
A9
A10
A11
A12
A13
A14

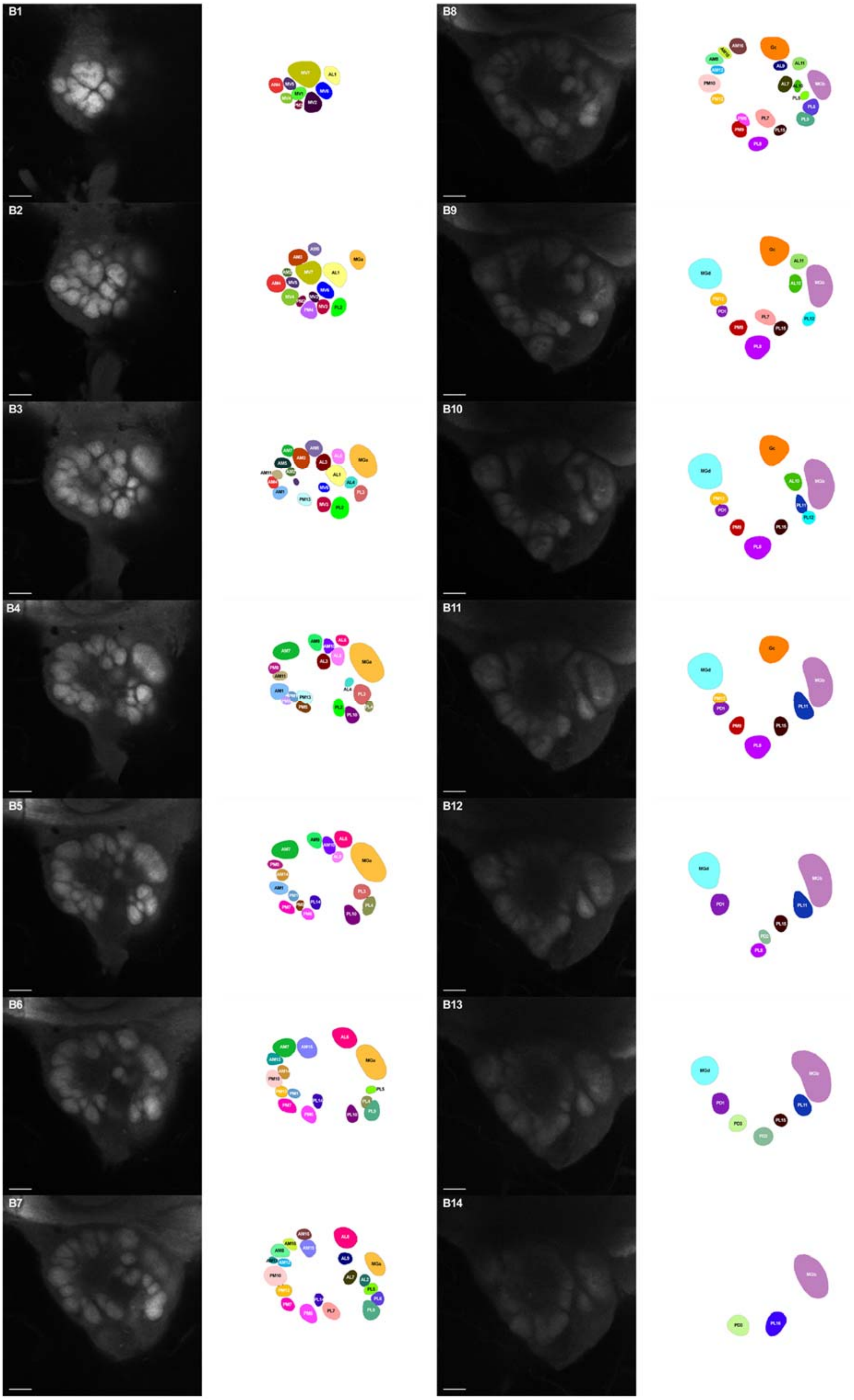
B1
B2
B3
B4
B5
B6
B7
B8
B9
B10
B11
B12
B13
B14

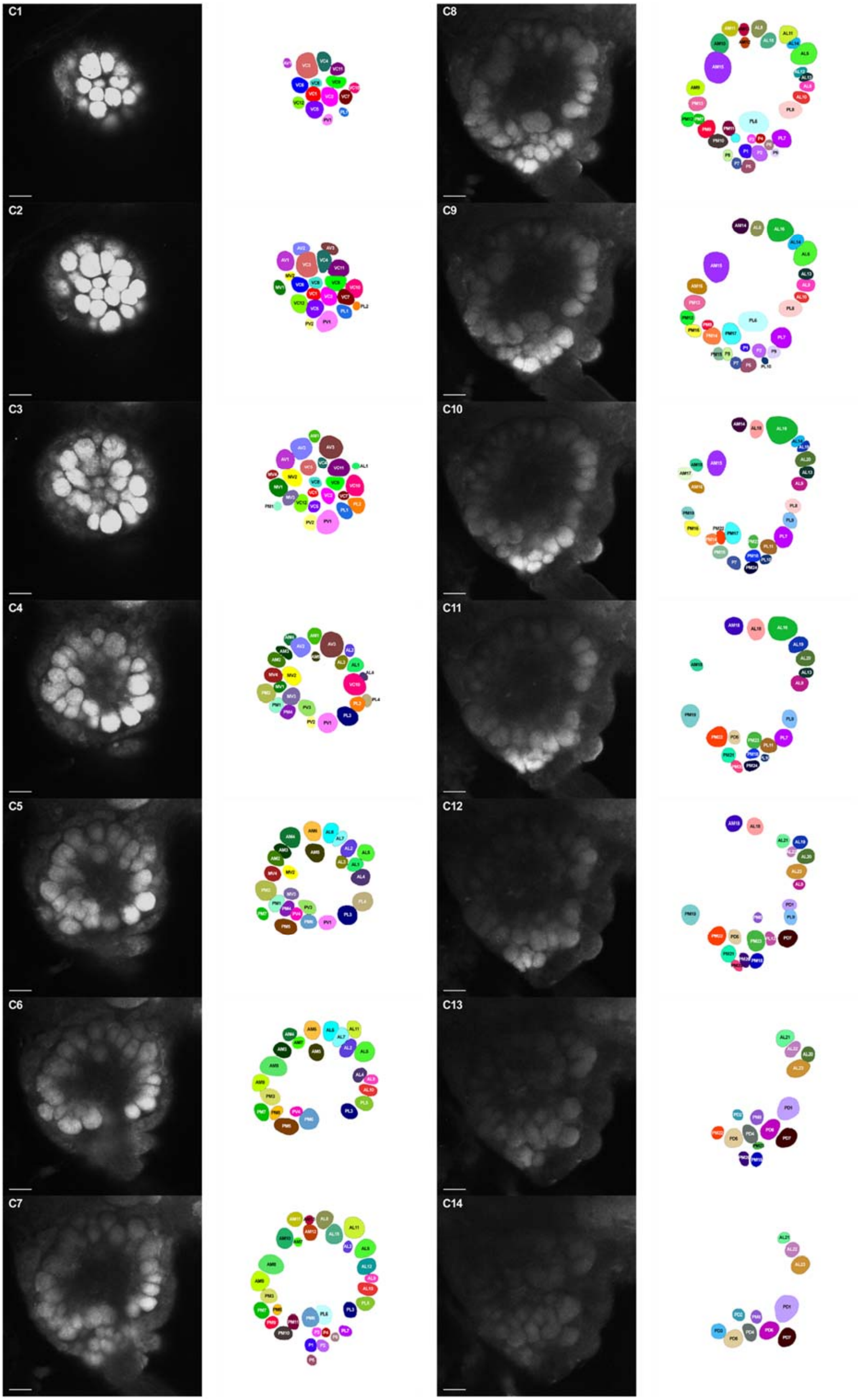
C1
C2
C3
C4
C5
C6
C7
C8
C9
C10
C11
C12
C13
C14

Figure S2 Confocal image and modelling of ALs in *A. cerana*: (A1-A14) queen, (B1-B14) drone, (C1-C14) worker. The anti SYNORF1 was used and Cy2 was used as a secondary antibody. Fourteen image stacks were selected from each caste. The left side is the original confocal image and the right side is the glomerulus identified and manually segmented using Amira software. Scale bar = 50 μm.
